# A Hybrid Occupational Risk Assessment of *Legionella pneumophila* in Hotel Water Systems Associated with TALD Cases

**DOI:** 10.3390/microorganisms14061257

**Published:** 2026-06-02

**Authors:** Antonios Papadakis, Vasileios Diamantopoulos, Eleftherios Koufakis, Anna Psaroulaki, Dimosthenis Chochlakis

**Affiliations:** 1 Department of Clinical Microbiology and Microbial Pathogenesis, School of Medicine, University of Crete, Voutes—Staurakia, 71110 Heraklion, Greece; psaroulaki@uoc.gr; 2 Department of Public and One Health, University of Thessaly, 43100 Karditsa, Greece; vdiamantop@uth.gr; 3 Directorate General of Public Health and Social Welfare of the Region of Crete, 71201 Heraklion, Greece; 4 Civil Protection Authority of the Region of Crete, 71201 Heraklion, Greece; elkoufakis@crete.gov.gr; 5 Regional Laboratory of Public Health of Crete, School of Medicine, 70013 Heraklion, Greece

**Keywords:** *Legionella pneumophila*, hotel water systems, occupational exposure, semi-quantitative risk assessment, job–exposure matrix, environmental surveillance, water safety plan, physicochemical deviations

## Abstract

Travel-associated Legionnaires’ disease (TALD) investigations in hotels have generated extensive environmental monitoring data. However, the occupational implications for workers who operate, maintain, clean, or inspect the same systems are rarely assessed. We developed a hybrid framework integrating a semi-quantitative environmental hazard model with deterministic Quantitative Microbial Risk Assessment (QMRA). In the first model, culture concentration bands were combined with physicochemical deviation indicators (temperature, free residual chlorine, and pH) to derive point-level hazard ($H_{i}$) and zone-level hazard (${\bar{H}}_{z}$). In the second model, a job-based presence matrix was combined with zone-specific serogroup-based severity using a simplified World Health Organization (WHO)-style 3 × 3 likelihood–severity approach. *Legionella pneumophila* (≥50 CFU/L) was detected in 29.94% of water samples and was significantly associated with low chlorine (<0.2 mg/L; RR 2.90) and hot water temperature < 55 °C (RR 3.07). To support comparative occupational exposure stratification, QMRA was applied to estimate the daily inhaled dose (d) for 15 worker groups, indicating variability in modeled biological exposure across occupational categories. Within this framework, modeled occupational exposure potential was shaped by the combined influence of pathogen concentration and assumed exposure duration. Under the hazard model, the highest zone-level hazard estimate was observed in kitchens and food and beverage (F&B) areas (${\bar{H}}_{z}$ = 2.607), followed by machinery rooms (${\bar{H}}_{z}$ = 2.022) and guest rooms (${\bar{H}}_{z}$ = 1.874). These findings support the integration of worker protection into water safety management, particularly in areas and groups overlooked in routine investigations.

## 1. Introduction

Legionnaires’ disease surveillance in Europe mainly records community-acquired, travel-associated, and healthcare-associated cases, while travel-associated Legionnaires’ disease (TALD) is monitored through the European Legionnaires’ Disease Surveillance Network (ELDSNet). TALD represents a significant component of the total burden of Legionnaires’ disease in Europe, with ELDSNet data showing that approximately 20% of reported cases are related to recent travel. Hotels, tourist resorts, and similar accommodation facilities are consistently identified as the settings most frequently involved in TALD clusters and related epidemiological investigations [1,2,3,4,5]. The detection of TALD clusters via ELDSNet triggers coordinated environmental surveys and preventive interventions in accommodation facilities across participating countries, making TALD a form of legionellosis subject to systematic surveillance. These investigations generate detailed environmental data, including temperature, pH, residual disinfectant levels, and culture results, from multiple points within hot- and cold-water distribution systems as well as recreational water networks [6,7,8]. European guidelines further emphasize that regular testing for *Legionella* and the implementation of appropriate control measures in artificial [9] water systems can prevent outbreaks and epidemics in tourist accommodations and other high-risk buildings. In contrast, there is currently no equivalent, harmonized, and systematic recording system for occupational legionellosis as a separate category, even at a global level. Consequently, the occupational dimension of exposure remains less visible in surveillance, although it may be particularly important in settings such as hotel water systems, where workers involved in operation, maintenance, inspection, or remediation may encounter the same contaminated networks that are primarily investigated in relation to guest safety [10].

Findings from occupational health and case investigations have shown that workers can become infected with *Legionella* in industrial facilities, office buildings, and healthcare facilities when performing maintenance work or aerosol-generating procedures in contaminated systems [11,12,13,14,15,16,17,18,19,20,21,22,23]. Systematic reviews have documented hundreds of confirmed occupational infections over several decades, including fatal outcomes, with most exposures related to cooling towers, industrial water circuits, and building networks [12,24,25,26,27]. However, few documented occupational cases originate from the hospitality sector, and only a limited number of hotel workers have been described in the literature, despite the recognized risk of *Legionella* in tourist accommodations [28,29]. This discrepancy suggests possible under-recognition or under-reporting of occupational Legionnaires’ disease in hotels and highlights the need for standardized assessment frameworks focused on worker exposure [30,31,32].

This need is reinforced by the hospitality environment itself, in which maintenance technicians, pool and spa staff, maids and cleaners, kitchen staff, external contractors, and sanitary inspectors routinely work in close proximity to showers, spa pools, decorative fountains, and other aerosol-generating water sources [33,34,35]. However, hotel workers are rarely the primary focus of *Legionella* investigations, and environmental data collected during TALD inspections are seldom translated into explicit indicators of occupational exposure by profession, task, or functional zone. Existing European and national *Legionella* control guidelines recognize workers and contractors as potential risk groups but mainly provide qualitative recommendations and do not incorporate semi-quantitative tools linking measured environmental conditions to occupational presence patterns. From an occupational safety perspective, this is important because higher-order preventive measures should take precedence over the reliance on personal protective equipment alone, consistent with the hierarchy of controls promoted by the Occupational Safety and Health Administration (OSHA) [36,37,38]. Despite this, occupational exposure in TALD-linked hotel environments is rarely translated into a structured worker-focused assessment framework, leaving an important gap between environmental investigation findings and practical occupational risk management.

Recreational water systems such as swimming pools, spa pools, and showers may also contribute to *Legionella* exposure when disinfection, circulation, or hygiene conditions deviate from the standards. Recent evaluations of hotel pools during COVID-19 have shown that pool hygiene and system performance can fluctuate significantly, indicating that the risk of *Legionella* in hospitality settings extends beyond hot-water systems to recreational environments [39,40,41,42,43]. These observations further support the need for a structured assessment of worker exposure in hotel environments.

Quantitative microbial risk assessment (QMRA) models have been used to estimate the risk to humans from environmental exposure to *Legionella* in settings such as whirlpool spas and hot water systems. Although these models can help relate environmental *Legionella* levels to infection risk, their use in hotel occupational settings remains limited, mainly because environmental findings are difficult to link to workers’ actual activities and because current guidance does not provide standardized tools for this purpose [44,45,46,47].

The present study was designed to address this gap by developing a hybrid framework for evaluating occupational risk related to *L. pneumophila* in hotel environments. The first component generated point- and zone-level environmental hazard estimates based on microbiological and physicochemical findings, whereas the second applied an independent World Health Organization (WHO)-style 3 × 3 approach to classify occupational risk by worker group and functional zone. In this way, the study extends TALD investigations beyond a purely TALD-focused environmental investigation framework and offers a practical and scalable basis for integrating worker protection into occupational risk assessments, *Legionella* risk assessments, and Water Safety Plans (WSPs) in hotel facilities. Future research should further refine this occupational prioritization framework by incorporating larger datasets, a wider range of facility types, and more detailed task-based exposure information. Advanced computational tools may support more consistent risk stratification and operational decision-making, but the present framework should not be interpreted as a clinically validated predictive model of infection [48].

While semi-quantitative methods provide a baseline for hazard identification, they often lack the resolution required to differentiate between roles with similar presence patterns but different biological exposure profiles. To bridge this gap, this study introduces a hybrid approach that complements traditional risk scoring with deterministic Quantitative Microbial Risk Assessment (QMRA). In the present study, the QMRA component was used as a deterministic exposure-modeling layer to support comparative occupational stratification across job categories, rather than as a full probabilistic model of infection risk. By estimating the daily inhaled dose (d)  through deterministic modeling, we provide a more granular evaluation of the risk across 15 distinct job categories, allowing for evidence-based prioritization of protective measures.

## 2. Materials and Methods

### 2.1. Study Framework and Environmental Data

The study included 648 water samples collected in 2025 from 25 hotels in the four Regional Units of Crete by Public Health inspectors of the Region of Crete and sent for microbiological analysis to the Regional Public Health Laboratory of the National Public Health Organization (NPHO). All participating hotels had an epidemiological link to at least one confirmed case of TALD during the 2024–2025 period, according to national and European surveillance definitions. Environmental surveys and sampling were carried out from January to November 2025, including hotels that were linked to TALD cases notified at the end of the 2024 tourist season but had already closed when the results of the inspection became available due to the end of seasonal operation. No data was collected on the health status of the workers, and their presence in the work zones was considered to comply with normal operational standards, regardless of TALD status. Water samples were collected from each point in a sterile plastic 1 L bottle containing a 0.1 N solution of sodium thiosulfate (Na_2_S_2_O_3_) (15.81 g/L in distilled water) in accordance with ISO 5667-1:2023 [49]. During sampling, the on-site water temperature (°C) was measured at each point. The free residual chlorine (mg/L) and pH were measured only in cold-water samples. These measurements were subsequently used to derive deviation indicators for semi-quantitative environmental hazard scoring. In addition, basic installation characteristics were recorded, such as the year of construction, the size of the unit (number of rooms and number of beds) and the star category. Temperatures were measured both immediately and after one minute of water flow for the hot network and two minutes of flow for the cold network. Furthermore, data were collected regarding the relative proximity of each room to the engine room (classified as close or distant) and the specific room where the associated patient had stayed when such information was available. The availability of room-specific case information depended on what could be operationally documented during the environmental investigation, in accordance with confidentiality requirements.

For the purposes of the study, the water samples collected were classified into five functional zones within each facility: (i) guest rooms, (ii) machinery rooms and water production and/or storage systems, (iii) kitchens and food and beverage (F&B) areas, (iv) gardens and outdoor plumbing/irrigation systems, and (v) recreational areas (swimming pools, spa/whirlpool/jacuzzi pools and associated showers). This functional categorization was based on the typical operational structure of hotels. Sampling points included, but were not limited to, shower outlets and bathroom taps in rooms, boiler outlets and return lines, solar thermal outlets, storage tank outlets, municipal water inlets, kitchen/restaurant/bar taps, ice machine water, garden taps, irrigation water, decorative fountains, and (where applicable) reclaimed water outlets, as well as pool/spa water and pool/spa/beach showers. A full mapping of sample types and functional zones is provided in Table S1. Water samples were obtained either as direct or indirect point samples, and room samples were taken from both the hot and cold distribution networks. Sampling was performed within the operational context of routine TALD-related environmental investigations. The selection of sampling points reflected the investigation needs, the structure of each hotel’s water system, and the functional relevance of each outlet at the time of inspection.

Microbiological analysis for *Legionella* was performed by the Regional Public Health Laboratory of Crete using culture methods according to ISO 11731:2017 [50], and concentrations were expressed in CFU/L. After collection, samples were transported to the Regional Public Health Laboratory of Crete and processed according to standard laboratory procedures for Legionella culture analysis. The laboratory detection/report limit was 50 CFU/L; values < 50 CFU/L were treated as below the detection/quantification limit and were not considered zero. Where available, laboratory reports included additional information for *L. pneumophila* serogroup 1, serogroups 2–14 and non-*pneumophila Legionella* species. All environmental and operational data were anonymized at the facility level.

### 2.2. Statistical Analysis and Facility-Level Risk Assessment

Facility-level risk characterization was based on available structural, operational, and physicochemical information recorded during environmental investigations. Statistical analyses were performed using IBM SPSS Statistics v30.0 (IBM Corp., Armonk, NY, USA) and Epi Info v7.2.7.0 (Centers for Disease Control and Prevention, Atlanta, GA, USA). Relative risk (RR) estimates were additionally cross-validated using the MedCalc online RR calculator Version 23.4.2 (MedCalc Software Ltd., Ostend, Belgium). Culture-based outcomes derived from *Legionella* CFU/L results (e.g., positivity thresholds and concentration bands) were calculated among processed samples only (*n* = 628), excluding samples not analyzed due to growth of flora (*n* = 20). Such growth is a recognized methodological limitation in culture-based *Legionella* detection, as excessive interfering flora can inhibit or prevent the recovery of *Legionella* spp. under ISO 11731 procedures [51,52,53,54,55].

Descriptive statistics are presented as frequencies and proportions with 95% confidence intervals (CIs). For proportions, 95% CIs were calculated using the Wilson score method; when sample sizes were very small, exact (Clopper–Pearson) 95% CIs were used. Associations between categorical variables were evaluated using the chi-square test (two-tailed, uncorrected) or Fisher’s exact test (two-tailed), as appropriate. Fisher’s exact test was applied when expected cell counts were small or when sparse 2 × 2 tables were present. Odds ratios (ORs), relative risks (RRs), 95% CIs, and absolute risk differences were calculated for selected risk factors. Statistical significance was set at *p* < 0.05, while *p* < 0.0001 was considered highly significant.

To comprehensively assess the robustness of all investigated exposure and contextual risk factors against potential unmeasured confounding, E-values were calculated for all analyzed parameters, following the framework proposed by VanderWeele and Ding [56]. The E-value represents the minimum strength of association that an unmeasured confounder would need to have with both the exposure indicator and *L. pneumophila* positivity, conditional on the measured covariates, to fully explain away the observed risk ratio to the null value (RR = 1). Lower-bound E-values, calculated from the lower limits of the 95% confidence intervals, were also systematically computed to assess the strength of unmeasured confounding that would be required to move the confidence interval to include the null and thereby alter the statistical significance of the findings [57].

### 2.3. Contamination Scoring

Each sample was assigned a contamination score based on the measured concentration of *L. pneumophila* (CFU/L). To align with widely recognized operational action levels, contamination scoring was delineated into three categories: (i) <1000 CFU/L (score 0), (ii) 1000 ≤ CFU/L < 10,000 (score 1), and (iii) ≥10,000 CFU/L (score 2). This 0–2 scale classifies concentrations into zones of increasing microbial load and facilitates interpretability using practically applied reference/action thresholds in environmental surveillance.

In addition, the 1000 CFU/L benchmark is consistent with the parametric value for *Legionella* used in the European drinking-water risk-management framework (Directive (EU) 2020/2184; Annex I, Part D) and with Greek legislation transposing the Directive (Ministerial Decision 27829/2023; Government Gazette 3525/B/25.05.2023), which specifies *Legionella* with a parametric value of 1000 CFU/L for monitoring in internal drinking-water distribution systems of priority premises, including tourist facilities and hotels. Although the parametric value refers to *Legionella* spp., it was used here as a pragmatic benchmark for categorizing the culture results for *L. pneumophila*.

Results reported as <50 CFU/L (laboratory LOD) were assigned to the <1000 CFU/L category for scoring, while remaining explicitly left-censored for descriptive analyses of culture concentrations.

Let $C_{i}$ denote the contamination score for sample i, defined as follows:

(1) $$C_{i}=\left\{\begin{array}{ll} 0, & \mathrm{if}\;\mathrm{CFU}/L<1000\\ 1, & \mathrm{if}\;1000\leq \mathrm{CFU}/L\leq 9999\\ 2, & \mathrm{if}\;\mathrm{CFU}/L\geq 10,000 \end{array}\right.$$

where CFU/L denotes the culture-based concentration of *L. pneumophila*, expressed in colony-forming units per liter.

The contamination score ${(C}_{i})$ was treated as an ordinal proxy of increasing microbial burden and was used for semi-quantitative comparison rather than as a linear representation of dose or infection probability.

### 2.4. Physicochemical Deviation Scoring

Operational compliance at each sampling point was evaluated using three physicochemical criteria widely applied in *Legionella* control programs: temperature outside control ranges (see below), insufficient free residual chlorine, and pH outside the operational band of 6.5–9.5. Each criterion was operationalized as a binary deviation flag.

For each sample $s_{i}$, the temperature deviation was defined conditionally based on sample type. A deviation was assigned if the hot-water temperature was <50 °C for hot samples or the cold-water temperature was >20 °C for cold samples. Thus, $T_{i}=1$ if the temperature criterion was not met; otherwise, $T_{i}=0$. For boiler endpoints, operational thresholds were stricter: a temperature deviation was assigned when the boiler outlet was <60 °C or when the boiler return was <50 °C, reflecting production and recirculation control targets.

The free residual chlorine and pH were measured only in cold-water samples. Let $I_{Cl,i}$ and $I_{pH,i}$ indicate availability (1 = measured, 0 = not measured). A chlorine deviation was assigned if the free residual chlorine was <0.2 mg/L when measured $\left(Cl_{i}=1\right)$. A pH deviation was assigned if pH < 6.5 or >9.5 $\left(pH_{i}=1\right)$.

A raw deviation count was then computed as

(2) $$D_{i}=T_{i}+Cl_{i}+pH_{i}$$

Because not all physicochemical parameters were measured at every sampling point, a normalization step was applied to allow comparability across samples. The number of available measurements was defined as

(3) $$m_{i}=1+I_{Cl,i}+I_{pH,i}$$

The normalized physicochemical deviation score was computed as

(4) $$P_{i}=3\times \frac{D_{i}}{m_{i}}$$

This approach makes it possible to compare samples with complete and partial physics composed of data more consistently while also reducing the bias introduced by missing field measurements. Physicochemical deviation scores were recalculated separately for each temperature-threshold scenario by redefining the temperature deviation flag $T_{i}$ accordingly.

### 2.5. Point-Level Environmental Hazard

A point-level environmental hazard index was computed for each sample i by combining the contamination score and physicochemical deviation score using a simple additive rule:

(5) $$H_{i}=C_{i}+P_{i}$$

With this formulation, $H_{i}$ ranged from 0 to 5, since $C_{i}$ ranged from 0 to 2 and $P_{i}$ ranged from 0 to 3. Higher values represent increased environmental hazard at the sampling point.

Equal weighting of microbiological contamination and physicochemical deviation was intentionally adopted to avoid over-parameterization and to maintain the transparency and operational usability of the model.

### 2.6. Zone-Level Hazard

Each sampling point was assigned to one of the five functional zones described in Section 2.1. For each zone z, the zone-level environmental hazard was computed as the arithmetic mean of the point-level hazard values across all samples in that zone:

(6) $${\bar{H}}_{z}=\frac{1}{n_{z}}\sum_{i\in z}H_{i}$$

where $n_{z}$ denotes the total number of samples collected in zone z.

The value ${\bar{H}}_{z}\;$ represents the average environmental hazard characteristic of each functional zone and was used for a comparative environmental assessment across functional zones within the first semi-quantitative model.

### 2.7. Worker Groups

Fifteen worker groups were defined: housekeeping workers, public-area cleaning workers, general maintenance workers, plumbing and HVAC workers, kitchen workers, spa and pool workers, lifeguards, gardeners and outdoor maintenance workers, security and general services workers, management and administrative workers, external plumbing/HVAC contractors, external pool/spa contractors, water treatment and chemical dosing workers, occupational/technical safety workers, and public health inspectors/environmental health officers. These categories corresponded to typical job profiles and did not refer to specific individuals. Table 1 provides an indicative mapping of departments, representative job positions, and the main potential sources of *Legionella* exposure relevant to these worker groups.

### 2.8. Zone Presence Weight

For each worker group g and functional zone z, a presence likelihood weight $W_{g,z}\;$ was assigned on a 0–3 scale, where 0 = absent, 1 = occasional presence, 2 = regular but not continuous presence, and 3 = frequent/usual presence. These weights were assigned by expert judgment based on typical hotel work practices, task allocation, operational responsibilities, and field experience from environmental health inspections in hotel settings. They were intended to represent typical occupational presence patterns at the group level and were not derived from formal time–motion studies, individual attendance records, or direct observational measurements. The full matrix of $W_{g,z}\;$ values is presented in Table S2.

### 2.9. WHO-Style 3 × 3 Likelihood–Severity Occupational Risk Model

In the second, independent model, a simplified WHO-style 3 × 3 likelihood–severity framework was applied for communication-oriented occupational risk classification. For each worker group g and functional zone z, the likelihood score was assigned directly from the presence-weight matrix presented in Table S2, where values ranged from 0 (absent) to 3 (frequent/usual presence). Severity was defined at the functional-zone level according to serogroup findings from the environmental dataset. Functional zones in which *L. pneumophila* serogroup 1 (SG1) was detected were assigned a severity score of 3, whereas zones with detections limited to serogroups 2–14 were assigned a severity score of 2. The occupational risk score for each worker group–zone combination was computed as follows:

(7) $$R_{g,z}=W_{g,z}\times S_{z}$$

where $R_{g,z}\;$ denotes the occupational risk score, $W_{g,z}\;$ the worker-group- and zone-specific likelihood score derived from Table S2, and $S_{z}\;$ the zone-specific severity score. The resulting scores were interpreted using a three-level color-coded scheme: low, moderate, and high risk. Specifically, scores of 0–2 were classified as low risk (green), scores of 3–4 as moderate risk (yellow), and scores of 6–9 as high risk (red), as shown in Table S3. This second model was intended as a simple and independent occupational prioritization tool and was not mathematically derived from the first semi-quantitative environmental hazard model. This communication-oriented model was intentionally designed as a simplified occupational prioritization tool and should not be interpreted as an estimate of individual infection probability.

### 2.10. Quantitative Exposure Modeling (QMRA)

To complement the semi-quantitative risk estimation, the daily inhaled dose (*d*) for each job profile was modeled using a deterministic Quantitative Microbial Risk Assessment (QMRA) framework. The dose d, representing the estimated number of Colony-Forming Units (CFU) inhaled per working shift, is determined using Equation (8):

(8) d = C × P × B × t

where d is the estimated daily inhaled dose (CFU/day), C is the mean concentration of *L. pneumophila* in the water of the specific functional zone (CFU/L), P is the aerosolization partition coefficient, conservatively estimated at 10^−6^ L/m^3^ based on literature for indoor water fixtures as a simplified partitioning assumption linking waterborne concentration to inhalation exposure in the absence of direct air sampling data [58,59], B is the average breathing rate for light occupational activity (0.013 m^3^/min) [46,58], and t  is the representative duration of exposure (min) derived from the presence index $W_{g,z}$. The transition from the semi-quantitative index $W_{g,z}\;$ to the temporal variable (t) was performed using deterministic modeling: $W_{g,z}\;$= 3 (frequent presence) was assigned t = 480 min (full shift), $W_{g,z}\;$= 2 was assigned *t* = 120 min, and $W_{g,z}\;$= 1 was assigned t = 30 min. To provide comparative context, the exponential dose–response model *P* = 1 − exp(−*r*·*d*) was applied to the estimated daily inhaled doses, using *r* = 5.99 × 10^−2^ CFU^−1^ for *L. pneumophila* inhalation [46,59]. Daily infection probabilities ranged from 2.4 × 10^−5^ (Security/General services) to 3.7 × 10^−4^ (Kitchen and Stewarding staff). Annualized estimates (220 working days) ranged from 0.5% to 7.9%, with kitchen-zone workers and spa therapists showing the highest values due to the maximum assumed shift duration (480 min). These figures are comparative modeling outputs under standardized assumptions and do not represent absolute clinical risk predictions at the individual level.

### 2.11. Personal Protective Equipment (PPE) Mapping

PPE recommendations were developed per task and worker group in alignment with the hierarchy of controls under Directive 2000/54/EC, treating PPE as a supplementary control measure when engineering and organizational measures do not fully eliminate exposure potential. PPE categories were operationalized across four domains relevant to *Legionella*-related tasks: (i) gloves, (ii) eye/face protection, (iii) waterproof protective clothing, and (iv) respiratory protection (FFP2/FFP3) for aerosol-generating activities. Minimum task-based PPE recommendations used to standardize the mapping process are summarized in Table S4.

### 2.12. Process Overview

**Figure 1:**
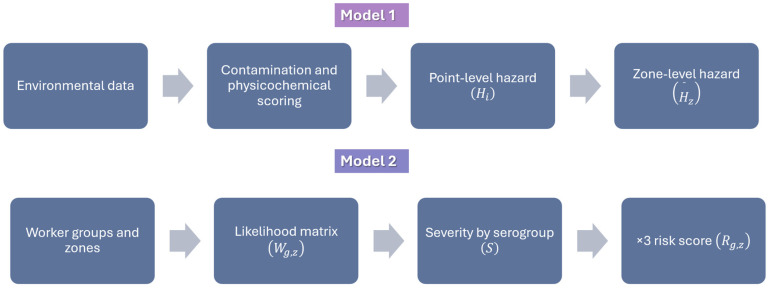
Overview of the two methodological models used in the study. Model 1 summarizes the semi-quantitative environmental hazard approach based on contamination severity and physicochemical deviations, resulting in point-level and zone-level hazard scores. Model 2 summarizes the independent WHO-style 3 × 3 occupational risk model based on worker-group- and zone-specific likelihood scores and serogroup-based severity.

The workflows of the two methodological models are summarized in Figure 1. Environmental monitoring data (temperature, free residual chlorine, pH, and culture-based microbiological results) were used to compute the point-level components (Equations (1)–(4)), which were then combined into the point-level environmental hazard index (Equation (5)) and aggregated to the zone-level hazard (Equation (6)). The first model generated a semi-quantitative environmental hazard characterization based on contamination severity and physicochemical deviations. In parallel, a second, independent model used worker-group- and zone-specific presence likelihood scores (Table S2) together with serogroup-based severity scores to compute a simplified WHO-style 3 × 3 occupational risk score (Equation (7)). The deterministic QMRA component was then applied as a complementary exposure-modeling layer to refine the comparison of worker groups under explicit operational assumptions. Accordingly, the three components were used in a sequential and complementary manner: the first to characterize environmental hazard, the second to prioritize occupational risk communication, and the third to provide a comparative estimate of modeled inhalation exposure.

## 3. Results

### 3.1. Sampling Profile and Investigated Points

A total of 648 water samples were collected from 25 TALD-linked hotels in Crete between January and November 2025. Of these, 20 samples did not yield reportable culture results because laboratory processing could not be completed due to extensive microbial flora growth. The mapping of each sample type to the corresponding functional hotel area is listed in Table S1. Sampling was concentrated on guest-room shower outlets, with room shower water accounting for 421/648 samples (64.97%; Wilson 95% CI: 61.22–68.54). Additional sampling included pool shower water (37/648, 5.71%; Wilson 95% CI: 4.17–7.77), boiler outlet water (32/648, 4.94%; Wilson 95% CI: 3.52–6.89), boiler return water (24/648, 3.70%; Wilson 95% CI: 2.50–5.45), municipal water inlets (22/648, 3.40%; Wilson 95% CI: 2.25–5.09), and water tank outlets (18/648, 2.78%; Wilson 95% CI: 1.76–4.35). Several additional sample types were collected at lower frequency, including kitchen sink tap water, garden irrigation water, ice-machine water, spa shower water, and fountain water.

### 3.2. Culture-Based Legionella spp. Concentration Bands (≥1000 and ≥10,000 CFU/L)

After excluding 20 samples affected by microbial flora, 628 samples were available for culture-based analysis. Using the predefined operational benchmarks for contamination scoring (Section 2.3), 80/628 processed samples (12.74%; Wilson 95% CI: 10.36–15.57) had *L. pneumophila* concentrations ≥ 1000 CFU/L, whereas 548/628 (87.26%; Wilson 95% CI: 84.43–89.64) had <1000 CFU/L. Within the ≥1000 CFU/L category, 49/628 samples (7.80%; Wilson 95% CI: 5.95–10.17) fell within the intermediate 1000–9999 CFU/L band, and 31/628 (4.94%; Wilson 95% CI: 3.50–6.92) reached ≥10,000 CFU/L, respectively.

### 3.3. Culture-Based L. pneumophila Positivity (≥50 CFU/L) by Sample Type

Across all processed samples, 188/628 (29.94%; Wilson 95% CI: 26.49–33.63) were positive for *L. pneumophila* at a concentration of ≥50 CFU/L. Non-*pneumophila Legionella* species were detected in 22/628 samples (3.50%; Wilson 95% CI: 2.32–5.25). Of these, 11 samples showed non-*pneumophila Legionella* without concurrent *L. pneumophila* detection, whereas 11 samples had concurrent detection of both non-*pneumophila Legionella* and *L. pneumophila*. Overall, *Legionella* detection (i.e., *L. pneumophila* and/or non-*pneumophila Legionella*) was recorded in 199/628 samples (31.69%; Wilson 95% CI: 28.17–35.43).

Serogroup findings were recorded at the sample level. In a subset of *L. pneumophila* positive samples, more than one serogroup was identified within the same sample. Thus, serogroup-specific counts represent the presence of each serogroup among the 188 *L. pneumophila* positive samples and are not mutually exclusive. The most frequently encountered serogroups were SG3 (*n =* 51), SG6 (*n =* 46), and SG8 (*n =* 42), followed by SG1 (*n =* 29), SG2 (*n =* 11), SG9 (*n =* 10), and SG7 (*n =* 9). Two additional detections were recorded under the combined “SG2–15” category, whereas no detections were reported for SG4, SG5, or SG10–SG15.

**Figure 2:**
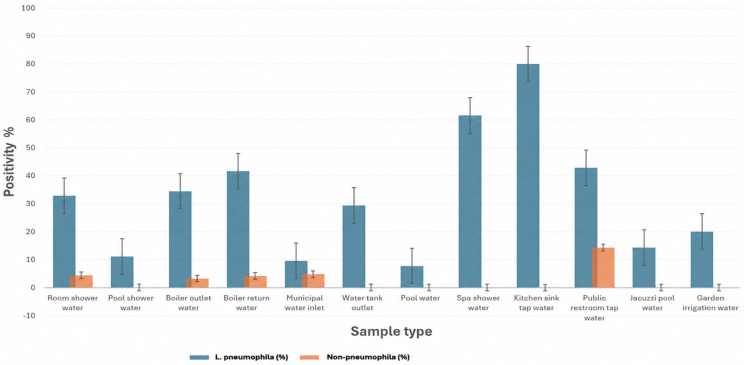
Culture positivity (≥50 CFU/L) by sample type among processed samples (*n =* 628). Bars show the proportion of processed samples positive for *L. pneumophila* and for non-*pneumophila Legionella* species at the ≥50 CFU/L threshold.

Culture positivity at ≥50 CFU/L varied by sample type. Room shower water, the dominant sampling category, showed overall *Legionella* positive findings in 143/408 samples (35.05%), while *L. pneumophila* positivity was observed in 134/408 (32.84%). Higher *L. pneumophila* positivity proportions were observed in several low-frequency categories, including kitchen sink tap water (8/10, 80.00%) and spa shower water (8/13, 61.54%). Boiler outlet and boiler return waters showed *L. pneumophila* positivity of 11/32 (34.38%) and 10/24 (41.67%), respectively, while pool shower water demonstrated lower *L. pneumophila* culture positivity (4/36, 11.11%). Figure 2 summarizes positivity patterns by sample type.

### 3.4. Association Analyses (RR) for L. pneumophila Positivity (≥50 CFU/L)

Associations between culture positivity for *L. pneumophila* (≥50 CFU/L) and selected physicochemical and structural parameters were evaluated using 2 × 2 contingency tables. Odds ratios (ORs), relative risks (RRs), risk differences, 95% confidence intervals (CIs), and two-tailed *p*-values were calculated and are presented in Table 2. Pearson’s χ^2^ test (uncorrected) was applied unless sparse cell counts were present, in which case Fisher’s exact test (two-tailed) was used. As summarized in Table 2, *L. pneumophila* positivity was most strongly associated with low free residual chlorine and inadequate hot-water temperature control. Free residual chlorine < 0.2 mg/L was significantly associated with positivity (RR = 2.90; 95% CI: 1.92–4.39), while hot-water temperature < 55 °C showed the strongest thermal association (RR = 3.07; 95% CI: 1.78–6.16). Hot-water temperature < 50 °C was also significantly associated with positivity, although with a more moderate effect size (RR = 1.54; 95% CI: 1.13–2.10). Star classification below 4 and sampling from the TALD case room were also significantly associated with *L. pneumophila* positivity. No significant associations were observed for the cold-water thresholds or the examined boiler temperature parameters. Sensitivity analyses via E-value profiling were systematically applied to all risk factors (Table 2). The strongest E-values were observed for hot-water temperature < 55 °C (E-value = 5.59; lower-bound E-value = 2.95) and free residual chlorine < 0.2 mg/L (E-value = 5.25; lower-bound E-value = 3.25), indicating that these operational parameters were comparatively robust to potential unmeasured confounding. The association for hot-water temperature < 50 °C showed lower but still meaningful robustness (E-value = 2.44; lower-bound E-value = 1.50). For structural and contextual markers that reached statistical significance, the calculated values indicated moderate robustness: star classification < 4 (RR = 1.89) demonstrated an E-value of 3.12, with a lower-bound E-value of 1.87, while samples originating from the TALD case room (RR = 1.48) showed an E-value of 2.32, with a lower-bound E-value of 1.49. For all non-statistically significant operational parameters, including cold-water thresholds and boiler measurements, the E-values were uniformly 1.00, indicating that no statistically significant effect size was present to be further attenuated by unmeasured confounding.

### 3.5. Environmental Hazard Scoring Outputs (Equations (1)–(6))

Applying the semi-quantitative scoring model to the 628 processed samples, the contamination score $C_{i}\;$ (Equation (1)) classified 548/628 (87.26%) samples in the <1000 CFU/L band (score 0), 49/628 (7.80%) in the 1000–9999 CFU/L band (score 1), and 31/628 (4.94%) in the ≥10,000 CFU/L band (score 2).

For physicochemical deviation scoring (Equations (2)–(4)), temperature measurements were available for 552/628 samples, free residual chlorine for 243/628, and pH for 143/628 samples. Among the recorded values, temperature deviation ($T_{i}=1$) occurred in 426/552 (77.17%) samples under the <55 °C scenario and in 344/552 (62.32%) samples under the <50 °C scenario, free residual chlorine < 0.2 mg/L ($Cl_{i}=1$) in 70/243 (28.81%) samples, and pH outside 6.5–9.5 ($pH_{i}=1$) in 3/143 (2.10%) samples. The availability of physicochemical measurements and the proportion of deviations among recorded values are summarized in Table S5. Because not all physicochemical parameters were recorded at every point, the normalized physicochemical deviation score $P_{i}$ was computed according to Equation (4), which scaled the observed deviation count to a common 0–3 range across samples with different numbers of available measurements.

Point-level environmental hazard scores $H_{i}=C_{i}+P_{i}\;$ (Equation (5)) were computable for 578/628 (92.04%) samples, with at least one recorded physicochemical parameter. Across these scored samples, $H_{i}\;$ had a mean of 1.908 (SD 1.381), a median of 2.0, and ranged from 0 to 5. The distribution of $H_{i}\;$ was as follows: 0 in 125/578 (21.63%), 1 in 111/578 (19.20%), 1.5 in 45/578 (7.79%), 2 in 24/578 (4.15%), 2.5 in 5/578 (0.87%), 3 in 224/578 (38.75%), 3.5 in 2/578 (0.35%), 4 in 25/578 (4.33%), and 5 in 17/578 (2.94%) scored samples.

**Figure 3:**
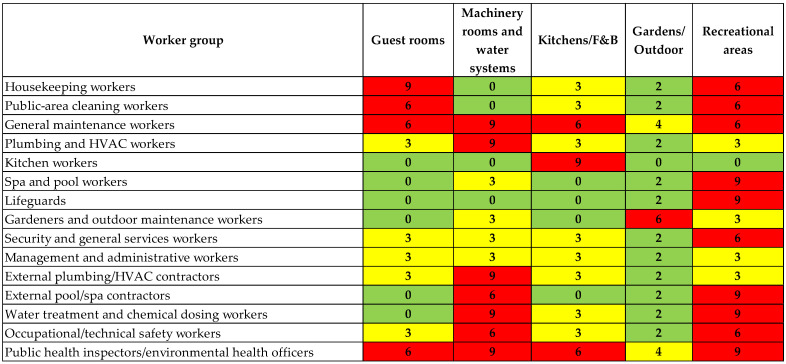
WHO-style 3 × 3 occupational risk profile by worker group and functional zone. Colors indicate risk category according to the predefined 3 × 3 matrix: green = low (0–2), yellow = moderate (3–4), and red = high (6–9).

Zone-level hazard ${\bar{H}}_{z}\;$(Equation (6)), calculated as the mean $H_{i}$ within each functional zone, was highest in kitchens and food and beverage areas (${\bar{H}}_{z}=2.607$, SD 1.130; scored n=14), followed by Machinery Rooms and Water Production/Storage Systems (${\bar{H}}_{z}=2.022$, SD 1.503; scored n=90), Guest Rooms (${\bar{H}}_{z}=1.874$, SD 1.404; scored n=405), Recreational Areas (Pools/Spa) (${\bar{H}}_{z}=1.825$, SD 1.097; scored n=63), and Gardens and Outdoor Plumbing/Irrigation (${\bar{H}}_{z}=1.750$, SD 0.758; scored n=6). As shown in Table 3 and Figure 3, the zone-level mean hazard ${\bar{H}}_{z}\;$ varied across functional areas, with the highest values observed in Kitchens and Food and Beverage Areas, followed by Machinery Rooms and Guest Rooms. Detailed zone-level environmental hazard values are additionally provided in Table S6.

### 3.6. WHO-Style 3 × 3 Occupational Risk by Worker Group and Functional Zone

Applying the independent WHO-style 3 × 3 occupational risk model, worker-group- and zone-specific risk scores were calculated as $R_{g,z}=W_{g,z}\times S_{z}$, where $W_{g,z}\;$ denotes the presence likelihood weight from Table S2 and $S_{z}\;$ the zone-specific severity score derived from serogroup findings. SG1 was detected in guest rooms, machinery rooms and water production/storage systems, kitchens and food and beverage areas, and recreational areas; these zones were therefore assigned a severity score of 3. In contrast, gardens and outdoor plumbing/irrigation systems were assigned a severity score of 2, as only serogroups 2–14 were detected in that zone.

As shown in Figure 3, the highest occupational risk scores were observed in worker-group and zone combinations characterized by frequent or usual presence in SG1-positive environments. High-risk scores were recorded for housekeeping workers in Guest Rooms, kitchen workers in Kitchens and Food and Beverage Areas, spa and pool workers and lifeguards in Recreational Areas, and multiple technical profiles in Machinery Rooms and Water Production/Storage Systems, including general maintenance workers, plumbing and HVAC workers, external plumbing/HVAC contractors, water treatment and chemical dosing workers, and public health inspectors/environmental health officers. Gardens and Outdoor Plumbing/Irrigation systems generally yielded lower scores because this functional area was assigned a lower severity level. Overall, the WHO-style 3 × 3 framework identified the most critical worker-group–zone combinations in guest-room, machinery-room, kitchen/F&B, and recreational-water settings.

### 3.7. Occupational Exposure Stratification Through Modeled Dose Estimation

To complement the semi-quantitative risk assessment, a deterministic QMRA was used to estimate the daily inhaled dose (d) for 15 distinct worker groups based on their presence in the identified functional zones. This hybrid approach allowed for a more granular differentiation of modeled exposure, especially in cases where presence patterns $\left(W_{g,z}\right)$ were similar but environmental hazard levels (${\bar{H}}_{z})\;$ differed. The results of this modeling are summarized in Table 4.

The modeling suggested that despite the traditionally lower aerosolization perception of kitchen environments, the combination of high pathogen concentrations in these zones and maximum exposure duration (480 min) may result in the highest estimated biological load for kitchen and stewarding staff members. In contrast, roles with high aerosol exposure potential but limited duration, such as public health inspectors during sampling, showed significant but lower cumulative daily doses. These estimates should be interpreted as comparative indicators of modeled occupational exposure potential under standardized assumptions, rather than as direct measures of clinical risk or predicted infection probability.

### 3.8. Task-Based PPE Recommendations for High-Exposure Activities

Following the occupational risk categorization presented in Table S3, task-based PPE recommendations were aligned with worker groups performing high-exposure or aerosol-generating activities in hotel water systems. Technical roles undertaking interventions in mechanical rooms and hot-water circuits (e.g., opening mechanical rooms after stagnation, outlet flushing, and repair work) were associated with the highest PPE requirements, including gloves, eye/face protection, waterproof clothing, and respiratory protection (FFP2/FFP3). Similarly, pool and spa system interventions involving filter backwashing, dosing adjustments, and draining/refilling procedures were mapped to PPE requirements, emphasizing splash protection and respiratory protection during aerosol-generating steps. For housekeeping and cleaning activities, baseline PPE (e.g., gloves) was considered sufficient for routine tasks, whereas escalation to respiratory protection was indicated for flushing or intensive work in settings with confirmed contamination or during corrective actions. The resulting worker-group- and task-specific PPE recommendations are listed in Table S7. These recommendations are intended to complement, rather than replace, higher-level control measures such as corrective disinfection, maintenance of hot-water and disinfectant control targets, outlet cleaning and descaling, controlled flushing procedures, restriction of access during remediation, and reduction in aerosol generation during high-exposure tasks.

## 4. Discussion

### 4.1. Principal Findings

This study transformed environmental investigation data from TALD-linked hotels into two structured semi-quantitative frameworks relevant to occupational health in hotel environments. The first model combined microbiological contamination bands for *L. pneumophila* with physicochemical deviation indicators to generate point and zone-level environmental hazard estimates. The second, independent model applied a simplified WHO-style 3 × 3 likelihood–severity approach to classify occupational risk across worker groups and functional zones. Together, these models indicated that, although technical and maintenance personnel remain important occupational groups for *Legionella*-related exposure assessment, worker categories with frequent or repeated presence in guest rooms, kitchens, and recreational-water settings may also warrant attention in occupational risk assessment. This prioritization is not presented as a separate legal category but as a practical way of operationalizing established occupational safety obligations. In the European context, these include biological-agent risk assessment, prevention or reduction in exposure, worker information and training, and the selection of appropriate collective and individual protective measures under Directive 2000/54/EC, together with the general preventive principles of Directive 89/391/EEC. In practical terms, occupational prioritization helps translate environmental findings into decisions about which worker groups, tasks, and hotel zones should receive earlier attention. For example, if a functional zone combines *L. pneumophila* detection, physicochemical deviation, and frequent worker presence, the framework can support prioritization of corrective sampling, technical inspection, outlet cleaning or descaling, controlled flushing procedures, task-specific training, and temporary escalation of PPE for workers performing aerosol-generating activities in that zone. More specifically, the identification of kitchens and food and beverage areas as a higher-hazard zone can guide hotel operators to verify hot-water control, inspect outlets, strengthen disinfection and maintenance procedures, and provide task-specific instructions to kitchen and stewarding staff, rather than applying only generic facility-wide recommendations.

In addition to supporting environmental and customer-focused risk management, the framework provides a structured basis for occupational prioritization that may assist in biological-agent risk assessment obligations under European occupational safety and health legislation. In this context, the deterministic QMRA component should be interpreted as a complementary exposure-stratification tool that adds granularity to the occupational comparison, rather than as a standalone predictive model of infection. In practical terms, the novelty of the framework lies in converting routine TALD-focused environmental investigation data into worker-relevant outputs that can support occupational prioritization within broader facility-level water safety management.

### 4.2. Environmental Determinants of L. pneumophila Positivity

Statistical analysis identified two main operational determinants of *L. pneumophila* positivity: insufficient disinfectant residual and inadequate hot-water temperature control. Low free residual chlorine (<0.2 mg/L) was strongly associated with positivity, underlining the importance of maintaining adequate disinfectant residuals to limit colonization in hotel water systems. Among the thermal indicators, hot-water temperature < 55 °C showed the strongest association with *L. pneumophila* positivity, whereas the <50 °C threshold remained statistically significant but with a more moderate effect size. This finding suggests that moderate loss of thermal control may already represent an important operational warning signal before more pronounced temperature failure occurs. In practical terms, relying only on the <50 °C threshold may underestimate early deterioration of hot-water control, while the <55 °C threshold may better capture conditions under which colonization becomes more likely in hotel water networks. In contrast, cold water > 20 °C and cold water > 25 °C were not significantly associated with positivity in this dataset, and neither boiler outlet temperature < 60 °C nor boiler return temperature showed statistically significant associations. These non-significant findings likely reflect the limited statistical power in the boiler-related strata and the influence of distal network conditions beyond the central plant measurements. Structural and contextual factors, including star classification below four and sampling from the room where the TALD case had stayed, were also associated with positivity, suggesting that both facility-level characteristics and local exposure-associated microenvironments may influence the likelihood of pathogen detection in TALD-related investigations. These findings are broadly consistent with previous hotel and building-water studies showing that insufficient disinfectant residual and inadequate thermal control are among the most important conditions favoring *Legionella* colonization. In Greek hotels, Kyritsi et al. reported significant associations between hotel water-system colonization and physicochemical parameters, supporting the relevance of routine environmental indicators in hospitality settings [26,60,61,62,63]. Borella et al. documented extensive colonization of Italian hotel hot-water systems, with *L. pneumophila* predominating, while more recent surveillance from tourist facilities in southern Italy again identified circulating water temperature as a major determinant of contamination [9,64]. In addition, previous work by Papadakis et al. in TALD-linked hotels and in recreational and garden areas of hotels has highlighted the importance of physicochemical control, residual disinfectant maintenance, and system management across multiple water-use environments, supporting the view that colonization risk in hospitality settings is shaped by combined operational and environmental determinants rather than by a single parameter in isolation [33,39,40,65]. More recent comparative analyses have likewise supported the relevance of low disinfectant residuals and suboptimal hot-water temperatures as key determinants of *Legionella* positivity across monitored water systems, reinforcing the threshold-based interpretation adopted in the present study [66,67,68]. Our findings therefore fit well within the broader literature, indicating that hotel water systems remain highly vulnerable when residual disinfectant is inadequate or thermal conditions are suboptimal. At the same time, the lack of a significant association for cold water > 20 °C suggests that distal conditions, stagnation, disinfectant failure, and local network characteristics may be more informative than a single cold-water threshold alone. The similarly non-significant result for cold water > 25 °C further supports the absence of a clear dose–response pattern for cold-water temperature in this dataset. For hot water, the stronger association observed at <55 °C compared with <50 °C, together with the absence of a significant association at <45 °C, suggests that early-to-moderate loss of thermal control may be more informative for operational prevention than more extreme temperature failure. This supports the use of higher preventive thresholds in hotel water safety management, particularly for distal outlets where temperature loss may indicate stagnation, insufficient circulation, or inadequate system balancing. This interpretation is also compatible with European TALD guidance, which emphasizes integrated system control, including temperature management, water circulation, and maintenance, rather than reliance on a single parameter in isolation. A further implication concerns risk communication, as *Legionella* surveillance findings may be misframed when isolated indicators are communicated without adequate system-level context [69]. The reported associations should also be interpreted within the analytical scope of the study, as they were primarily intended to identify operationally relevant environmental signals rather than to provide fully adjusted causal estimates.

### 4.3. Zone-Level Hazard Patterns and Operational Implications

Zone-level hazard varied across functional areas, with the highest values observed in kitchens and food and beverage areas, followed by machinery rooms, guest rooms, recreational areas, and gardens/outdoor plumbing/irrigation. This ranking suggests that environmental hazards in TALD-linked hotels are not confined to conventional guest rooms or spa-associated settings but may also emerge in operational service areas where water use, temperature deviations, and physicochemical non-compliance coexist.

Although Kitchens/F&B displayed the highest mean hazard, this estimate was derived from a comparatively small number of scored samples and should therefore be interpreted as an operational signal warranting targeted verification and denser sampling rather than as a stable facility-wide estimate. Likewise, the low-scoring sample count for Gardens/Outdoor Plumbing/Irrigation limits the precision of the corresponding zone-level estimate. Recreational areas showed a lower mean hazard than some indoor functional zones; however, this likely reflects the protective effect of autonomous and continuously regulated disinfection systems commonly used in pools and spa facilities, rather than the absence of risk. Operational failures in circulation, disinfectant residuals, or maintenance practices may still create conditions favorable for *Legionella* persistence in these systems.

At the point level, scored samples were distributed across a broad hazard range, indicating that microbiological burden and physicochemical deviations can cluster in specific locations even within the same hotel environment. Stratifying risk by occupational zone offers a more practical approach to prevention because, rather than considering the hotel as a single occupational exposure environment, occupational and environmental controls can be tailored to the specific characteristics of each functional area. Direct comparison with previous occupational risk studies is limited, as most published studies in hotel environments have focused either on the overall colonization of the water system, on physicochemical risk factors, or on specific subsystems such as hot water distribution systems and recreational water facilities, rather than systematically classifying hotel functional zones according to average environmental risk. Nevertheless, the study findings are generally in line with the broader literature, which indicates that the risk of *Legionella* in hotels is not limited to room water supplies but can occur in multiple operational environments within the same facility. Therefore, the present framework helps bridge an important practical gap between TALD investigation data, environmental interpretation, and structured occupational prioritization in hotels by translating environmental findings into zone-level operational and worker-relevant risk prioritization, an area that remains only sparsely addressed in the existing hotel-based *Legionella* literature [56,70,71,72,73,74].

### 4.4. Occupational Exposure Profiles: Who Is at Risk and Where

The second, independent model used worker-group- and zone-specific presence likelihood weights together with zone-specific serogroup-based severity to generate a simplified WHO-style 3 × 3 occupational risk classification. This approach showed that occupational risk is shaped jointly by work patterns and microbiological severity within each functional zone. Guest Rooms, Machinery Rooms and Water Production/Storage Systems, Kitchens and Food and Beverage Areas, and Recreational Areas were assigned higher severity because SG1 was detected in these zones, whereas Gardens/Outdoor Plumbing/Irrigation systems were assigned lower severity because only serogroups 2–14 were detected there.

High-risk scores were recorded for housekeeping workers in Guest Rooms, kitchen workers in Kitchens and Food and Beverage Areas, spa and pool workers and lifeguards in Recreational Areas, for multiple technical profiles in Machinery Rooms and Water Production/Storage Systems, including general maintenance workers, plumbing and HVAC workers, external plumbing/HVAC contractors, water treatment and chemical dosing workers, and public health inspectors/environmental health officers. Gardens and Outdoor Plumbing/Irrigation systems generally yielded lower occupational risk scores because of their lower severity classification. Overall, the model identified the most critical worker-group–zone combinations in guest-room, machinery-room, kitchen/F&B, and recreational-water settings.

These findings reinforce an important occupational health message: in TALD-linked hotel environments, the relevant exposure potential extends beyond plant-room specialists and includes worker groups whose routine duties involve repeated contact with water outlets, wet environments, or aerosol-generating systems.

### 4.5. Bridging the Gap: From Hazard Scoring to Modeled Dose Estimation

Although semi-quantitative frameworks successfully identified high-hazard zones, they inherently lacked the resolution to account for the cumulative effects of prolonged exposure to environments with lower perceived aerosolization, such as kitchens. The integration of deterministic Quantitative Microbial Risk Assessment (QMRA) addressed this limitation by evaluating the daily inhaled dose (d) and estimating comparative infection probabilities as a function of duration and pathogen load. This modeling layer was used to refine relative exposure ranking across worker groups under explicit simplifying assumptions, not to infer absolute infection probabilities. This finding suggests that traditional risk models focusing primarily on aerosol-generating fixtures may overlook significant exposure profiles in hotel operational zones, where extended presence and high-water contamination coexist.

### 4.6. Integration with Water Safety Plans and the Hierarchy of Controls

The proposed framework is compatible with hotel Water Safety Plans because it translates routine environmental monitoring findings into structured hazard and occupational prioritization outputs. This is in line with guidance from the WHO, the Centers for Disease Control and Prevention (CDC), and the European Centre for Disease Prevention and Control (ECDC), all of which recommend building- and system-specific Water Safety Plans or water management programs that identify critical risk points and support the routine monitoring of *Legionella* control measures [75,76,77,78]. The strong association between inadequate disinfectant residuals and *L. pneumophila* positivity further highlights the need for continuous monitoring, timely corrective dosing, and regular operational verification of water quality. Likewise, the temperature association supports maintaining hot-water control targets and preventing distal cooling in distribution networks, in line with guidance emphasizing thermal control and operational monitoring as core elements of prevention [79].

From an occupational perspective, these measures represent higher-level controls within the hierarchy of controls, particularly engineering, operational, and work-practice controls. PPE should be treated as a supplementary layer for aerosol-generating tasks or direct interventions in suspected or contaminated systems, rather than as a substitute for water-system control. This approach is also consistent with the guidance from the OSHA, according to which personal protective equipment functions as the last line of defense and should not replace feasible engineering or administrative controls [80]. From a practical perspective, the framework may support prioritization of follow-up sampling, targeted technical controls, task-specific PPE escalation, and focused staff training in the hotel zones identified as higher hazard. In this respect, the framework does not replace existing Water Safety Plans or occupational health approaches but rather complements them by translating routine environmental findings into a more explicit worker-oriented prioritization process. From an intervention perspective, the framework can also help translate prioritization outputs into practical control measures. Corrective actions should first target source control and reduction in aerosol generation before reliance on PPE. Examples include maintaining hot-water circulation and distal outlet temperatures within control targets, ensuring adequate disinfectant residuals, cleaning and descaling showerheads and taps, applying corrective disinfection when contamination is detected, and performing targeted re-sampling after corrective actions. For activities such as flushing showers or outlets after stagnation, aerosol generation can be reduced by directing water flow straight to the drain, for example, through temporary hoses, outlet sleeves, or shower bags where operationally feasible. During remediation or high-risk maintenance activities, access to affected areas may need to be restricted, and staff involved in flushing, sampling, descaling, repair work, or other aerosol-generating tasks should receive task-specific instructions and appropriately scaled PPE when exposure cannot be adequately controlled by engineering or organizational measures alone.

### 4.7. Strengths, Limitations, and Interpretation

A major strength of this study is the use of real-world environmental investigation data from TALD-linked hotels and a transparent framework that can be replicated by public health services and hotel operators in the future. This study also integrates microbiological, physicochemical, and occupational dimensions in a structured manner that supports practical prioritization. This study has several limitations. First, the presence weights were expert-informed and did not capture individual-level time–activity patterns. They should therefore be interpreted as structured occupational approximations at the group level rather than direct behavioral measurements. Furthermore, quantitative dose estimates rely on literature-derived constants (aerosolization partition coefficient and breathing rate) ((P) and (B)) and environmental water concentrations, rather than direct air sampling or personalized dosimetry [46,58]. Second, physicochemical measurements, particularly for chlorine and pH, were not available for all samples. To address this, the physicochemical deviation score was normalized according to the number of available measurements; however, missing data may still limit precision in some locations. Third, zone-level hazard estimates depend on the number and representativeness of samples scored per zone, and functional areas with small denominators should be interpreted cautiously. Fourth, the occupational risk model was intentionally kept simple for communication purposes, while the addition of a deterministic QMRA layer helped provide a more detailed picture of potential exposure. However, this approach does not estimate the probability of infection at the individual level. The semi-quantitative hazard score should likewise be interpreted as an operational prioritization construct and not as a clinically validated predictive scale. Similarly, the broader framework should be regarded as a structured occupational prioritization and exposure-stratification framework, rather than as a formally validated predictive instrument. It is not merely a data-processing workflow, because it integrates environmental measurements, physicochemical deviations, worker presence assumptions, serogroup-based severity, and deterministic exposure modeling into worker-relevant prioritization outputs. However, it does not predict individual infection probability, clinical disease occurrence, or future outbreak risk. The study also did not include guest-level exposure data, guest testing, or individual clinical information beyond the epidemiological linkage that triggered the environmental investigation. Therefore, the framework should not be interpreted as an assessment of guest infection risk. Its guest-related relevance is limited to the use of TALD-focused environmental investigation data to support facility-level water safety management and worker-oriented occupational prioritization. The framework was developed and applied specifically to *L. pneumophila* because the scoring thresholds, serogroup-based severity assumptions, and QMRA parameters were based on *Legionella*-specific environmental and exposure considerations. Although the general structure could be adapted to other waterborne or aerosol-transmitted biological agents, such adaptation would require pathogen-specific concentration thresholds, exposure assumptions, dose–response evidence where available, and severity criteria. In addition, the study reflects investigation-time environmental sampling and does not capture temporal variability within the same water systems across seasons or repeated sampling rounds.

## 5. Conclusions

This study demonstrates that workers in hotel environments may experience meaningful *L. pneumophila* exposure potential across multiple departments, including maintenance and technical services, housekeeping, kitchens, gardens, spa and pool operations, external contractor activities, and public health inspection functions. By combining a semi-quantitative environmental hazard model derived from routine microbiological and physicochemical monitoring with an independent, WHO-style 3 × 3 occupational risk model based on worker presence and zone-specific severity, the proposed framework provides a structured and reproducible approach for prioritizing occupational risk in hotel water systems. The finding that hot-water temperature < 55 °C showed a stronger association with *L. pneumophila* positivity than the <50 °C threshold further supports the value of preventive thermal monitoring before substantial temperature failure becomes evident. Although developed in TALD-linked hotel settings, the framework may also provide a useful basis for adaptation to other building types where worker exposure to aerosolized water systems is relevant.

Integrating this occupational dimension into Water Safety Plan workflows and environmental investigation protocols may support more targeted prevention through system-level control measures, task planning, staff training, and appropriately scaled protective measures for high-exposure activities. The findings also suggest that areas such as kitchens and food and beverage settings, which are often underemphasized in routine hotel water monitoring, may warrant closer attention in future investigations and surveillance protocols.

This study supports a strategic transition from point-of-use monitoring to integrated zone-based management. By adopting this hybrid assessment framework, hotel operators can identify occupational groups with elevated exposure potential, such as kitchen and maintenance staff, ensuring that occupational health is not overlooked in routine water safety management and environmental investigations. Reducing pathogen concentration (C) at the zone level remains a key strategy for lowering the cumulative modeled inhaled dose (d)  for the entire workforce.

## Figures and Tables

**Table 1:** Hotel worker groups with potential *Legionella* exposure by department/role and main potential exposure sources.

Department	Job Position	Main Potential Sources of Exposure
Engineering/Maintenance	General maintenance technician	Hot and cold-water systems, guest room showers, mechanical rooms, pumps, valves
Engineering/Maintenance	Plumbing technician	Pipework repairs, showerheads and taps, removal of scale, stagnant sections of the network
Engineering/Maintenance	HVAC/cooling systems technician	Cooling towers, evaporative condensers, humidifiers, air-handling units (where present)
Engineering/Maintenance	Pool and spa systems technician	Swimming pools, spa pools, whirlpools, filters, backwash systems, dosing equipment
Spa/Wellness/Pool	Spa therapist/spa attendant	Spa pools, whirlpools, hydro-massage facilities, steam rooms, experience showers
Spa/Wellness/Pool	Pool attendant/lifeguard	Swimming pools, children’s pools, poolside showers, water features
Spa/Wellness/Pool	Spa receptionist/spa assistant	Presence in spa area with aerosol-generating facilities
Housekeeping/Floors	Room attendant (housekeeper)	Guest room showers and bathrooms, flushing of outlets during cleaning
Housekeeping/Floors	Public area cleaner	Public toilets, changing rooms, spa/fitness showers, staff showers
Housekeeping/Floors	Floor supervisor	Periodic presence in rooms and corridors with access to bathrooms
Food and Beverage (F&B)	Kitchen staff (chefs, cooks)	Hot water outlets in kitchens, dishwashing areas, steam and aerosols
Food and Beverage (F&B)	Stewarding/dishwashing staff	Dishwashers, sinks, hot water sprays and aerosols
Food and Beverage (F&B)	Waiters, bartenders	Ice machines, beverage dispensers connected to the water supply
Gardens/Exterior maintenance	Gardener/landscaping staff	Irrigation sprinklers, outdoor misting systems, decorative ponds and fountains
Gardens/Exterior maintenance	Exterior maintenance worker	Outdoor decorative fountains, waterfalls, façade cleaning systems
Front office/Administration	Receptionists/front-desk agents	General indoor air, lobby air-conditioning systems
Front office/Administration	Management, back-office staff	Offices supplied by central HVAC
Security/General services	Security guards	Patrols in all areas, including technical rooms and roofs with cooling systems
Security/General services	General services/handyman	Minor repairs in bathrooms, public areas, back-of-house facilities
External contractors	External pool and spa maintenance	Targeted work on pools, spa systems, filters, dosing equipment
External contractors	External HVAC/refrigeration technicians	Cooling towers, chillers, evaporative cooling systems
External contractors	Cleaning companies (outsourced housekeeping, etc.)	Cleaning of rooms and wet areas, similar tasks to in-house housekeeping
External contractors	Construction/refurbishment contractors	Work on water networks, tanks and spa facilities during renovations (short-term but intense exposure)
External/regulatory/safety	Public health inspectors/environmental officers	Inspections and sampling in mechanical rooms, plant rooms, recreational water systems and other high-risk water installations
External/regulatory/safety	Occupational/technical safety specialists	Risk assessments and audits in plant rooms, technical areas and high-risk installations

**Table 2:** Association of selected parameters with *L. pneumophila* positivity (≥50 CFU/L).

Risk Factors	Odds Ratio	95% CI	R.R.	95% CI	Risk Difference (%)	95% CI	*p* Value *	E-Value (Lower CI)
Free residual chlorine < 0.2 mg/L	4.59	2.48–8.50	2.90	1.92–4.39	30.84	17.84–43.84	<0.0001 (χ^2^)	5.25 (3.25)
Cold water > 20 °C	2.18	0.26–18.41	1.87	0.30–11.57	12.36	−14.09–38.80	0.6799 (Fisher)	1.00 (1.00)
Cold water > 25 °C	1.23	0.72–2.10	1.17	0.79–1.73	4.01	−6.27–14.29	0.4988 (Fisher)	1.00 (1.00)
Hot water < 45 °C	1.55	0.87–2.77	1.31	0.93–1.85	10.37	−3.51–24.25	0.1739 (Fisher)	1.00 (1.00)
Hot water < 50 °C	2.14	1.27–3.55	1.54	1.13–2.10	18.25	6.28–30.23	0.0038 (χ^2^)	2.44 (1.50)
Hot water < 55 °C	4.50	2.20–9.29	3.07	1.78–6.16	23.82	14.53–33.07	<0.0001 (χ^2^)	5.59 (2.95)
Boiler outlet temp. < 60 °C	2.25	0.38–13.35	1.76	0.47–6.62	16.91	−16.79–50.61	0.4414 (Fisher)	1.00 (1.00)
Boiler return temp. < 50 °C	2.70	0.51–14.37	1.77	0.67–4.71	23.78	−14.89–62.45	0.4081 (Fisher)	1.00 (1.00)
Star classification < 4	2.33	1.43–3.80	1.89	1.28–2.80	15.64	7.92–23.36	0.0004 (Fisher)	3.12 (1.87)
Sample from TALD case room	1.85	1.17–2.95	1.48	1.12–1.96	14.15	3.22–25.09	0.0104 (Fisher)	2.32 (1.49)

* *p* value: Pearson’s χ^2^ test (two-tailed, uncorrected) unless sparse data were present; for sparse tables, Fisher’s exact test (two-tailed) was used.

**Table 3:** Zone-level environmental hazard scores by functional area under the semi-quantitative environmental hazard model.

Functional Area	Scored *n*	Mean $H_{i}$/Zone-Level Hazard ${\bar{H}}_{z}$	SD
Kitchens and Food and Beverage Areas	14	2.607	1.130
Machinery Rooms and Water Production/Storage Systems	90	2.022	1.503
Guest Rooms	405	1.874	1.404
Recreational Areas (Pools/Spa)	63	1.825	1.097
Gardens and Outdoor Plumbing/Irrigation	6	1.750	0.758

**Table 4:** Modeled daily inhaled dose (expressed in scientific notation) and risk stratification across 15 hotel worker groups.

Worker Group	Primary Exposure Zone	Presence Index (*W_g,z_*)	Estimated Daily Inhaled Dose (*d*, CFU/day)	Estimated Daily Infection Probability (Pdaily)	Estimated Annual Probability (Pannual, 220 d)
Kitchen staff (chefs/cooks)	Kitchens/F&B	3	6.2 × 10^−3^	3.7 × 10^−4^	7.9 × 10^−2^
Stewarding/dishwashing	Kitchens/F&B	3	6.2 × 10^−3^	3.7 × 10^−4^	7.9 × 10^−2^
Spa therapist/attendant	Recreational Areas	3	5.1 × 10^−3^	3.1 × 10^−4^	6.5 × 10^−2^
Plumbing technician	Machinery Rooms	2	3.1 × 10^−3^	1.9 × 10^−4^	4.0 × 10^−2^
HVAC technician	Machinery Rooms	2	3.1 × 10^−3^	1.9 × 10^−4^	4.0 × 10^−2^
Pool/Spa technician	Recreational Areas	2	2.8 × 10^−3^	1.7 × 10^−4^	3.6 × 10^−2^
Room attendant (housekeeper)	Guest Rooms	3	2.5 × 10^−3^	1.5 × 10^−4^	3.3 × 10^−2^
General maintenance	All zones (weighted mean)	3	2.2 × 10^−3^	1.3 × 10^−4^	2.9 × 10^−2^
Public area cleaner	Public and spa shower areas	3	2.1 × 10^−3^	1.3 × 10^−4^	2.7 × 10^−2^
Public health inspectors	High-exposure sampling points	1	1.5 × 10^−3^	9.0 × 10^−5^	2.0 × 10^−2^
Lifeguard	Recreational Areas	3	1.1 × 10^−3^	6.6 × 10^−5^	1.4 × 10^−2^
Occupational safety specialist	Technical service areas	1	0.9 × 10^−3^	5.4 × 10^−5^	1.2 × 10^−2^
External HVAC contractors	Machinery Rooms	1	0.8 × 10^−3^	4.8 × 10^−5^	1.1 × 10^−2^
Gardener/landscaping	Gardens/Irrigation	2	0.8 × 10^−3^	4.8 × 10^−5^	1.1 × 10^−2^
Security/General services	General hotel service areas	1	0.4 × 10^−3^	2.4 × 10^−5^	0.5 × 10^−2^

Abbreviation: Annual infection probability was calculated assuming 220 working days per year. These values represent comparative modeling estimates under standardized assumptions and should not be interpreted as absolute clinical risk predictions.

## Data Availability

The data presented in this study are available on request from the corresponding author. The data is not publicly available due to privacy and confidentiality restrictions.

## Supplementary Materials

The following supporting information can be downloaded at https://www.mdpi.com/article/10.3390/microorganisms14061257/s1, Table S1: Mapping sample types to functional hotel areas. Table S2: Presence likelihood weights ($W_{g,z}$) by worker group and functional zone (0–3 scale). Table S3: WHO-style 3 × 3 occupational risk matrix based on likelihood and severity scores. Table S4: Minimum task-based personal protective equipment (PPE) recommendations by worker group. Table S5: Availability of physicochemical measurements and proportion of deviations among recorded values for temperature ($T_{i}$), free residual chlorine ($Cl_{i}$), and pH ($pH_{i}$). Table S6: Zone-level environmental hazard scores by functional area under the semi-quantitative environmental hazard model. Table S7: Task-based personal protective equipment (PPE) recommendations for worker groups performing high-exposure or aerosol-generating activities in hotel water systems.

## Abbreviations

The following abbreviations are used in this manuscript:

TALD	Travel-associated Legionnaires’ disease
WHO	World Health Organization
NPHO	National Public Health Organization
OSHA	Occupational Safety and Health Administration
PPE	Personal protective equipment
RR	Risk ratio
CI	Confidence interval
CFU/L	Colony-forming units per liter
SG	Serogroup
HVAC	Heating, ventilation, and air conditioning
F&B	Food and beverage
CDC	Centers for Disease Control and Prevention
ECDC	European Centre for Disease Prevention and Control
