# Supplementary material for: A Hybrid Occupational Risk Assessment of Legionella pneumophila in Hotel Water Systems Associated with TALD Cases
Source: Microorganisms. 2026 Jun 2;14(6):1257. doi: 10.3390/microorganisms14061257 (PMC13303895; doi:10.3390/microorganisms14061257)
Supplement: Supplementary file 1 [file microorganisms-14-01257-s001.zip › microorganisms-4305267-supplementary.pdf]

microorganisms

MDPI

Supplementary

Table S1. Mapping sample types to functional hotel areas.

Sample Type	Assigned Functional Area
Guest-room shower outlet	Guest rooms
Guest-room bathroom tap	Guest rooms
Boiler outlet water	Machinery rooms & water systems
Boiler return line	Machinery rooms & water systems
Solar heater outlet	Machinery rooms & water systems
Water storage tank outlet	Machinery rooms & water systems
Municipal inlet water	Machinery rooms & water systems
Kitchen tap	Kitchens & F&B
Restaurant bar tap	Kitchens & F&B
Ice-machine water	Kitchens & F&B
Garden tap	Gardens & exterior systems
Irrigation system water	Gardens & exterior systems
Decorative fountain water	Gardens & exterior systems
Reclaimed water outlet	Gardens & exterior systems
Swimming pool water	Recreational areas
Spa pool / whirlpool water	Recreational areas
Jacuzzi pool water	Recreational areas
Poolside shower	Recreational areas
Spa shower	Recreational areas
Beach / exterior shower	Recreational areas

Abbreviations: F&B, food and beverage

Table S2. Presence likelihood weights ($W_{g,z}$) by worker group and functional zone (0–3 scale).

Worker group	Guest rooms	Machinery rooms and water systems	Kitchens/F&B	Gardens/Outdoor	Recreational areas
Housekeeping workers	3	0	1	1	2
Public-area cleaning workers	2	0	1	1	2
General maintenance workers	2	3	2	2	2
Plumbing and HVAC workers	1	3	1	1	1
Kitchen workers	0	0	3	0	0
Spa and pool workers	0	1	0	1	3
Lifeguards	0	0	0	1	3
Gardeners and outdoor maintenance workers	0	1	0	3	1
Security and general services workers	1	1	1	1	2
Management and administrative workers	1	1	1	1	1
External plumbing/HVAC contractors	1	3	1	1	1
External pool/spa contractors	0	2	0	1	3
Water treatment and chemical dosing workers	0	3	1	1	3

Occupational/technical safety workers	1	2	1	1	2
Public health inspectors/environmental health officers	2	3	2	2	3

Weights represent typical presence frequency determined by task patterns in each functional zone: 0 = absent, 1 = occasional, 2 = regular, 3 = frequent/usual. These weights were used directly as likelihood scores in the second, independent occupational risk model. They are expert-informed and intended for comparative assessment rather than direct time-activity measurement.

Table S3. WHO-style 3x3 occupational risk matrix based on likelihood and severity scores.

Likelihood score ($L_{g,z}$)	Severity = 2 (SG2-14)	Severity = 3 (SG1)
0	0 - Low (Green)	0 - Low (Green)
1	2 - Low (Green)	3 - Moderate (Yellow)
2	4 - Moderate (Yellow)	6 - High (Red)
3	6 - High (Red)	9 - High (Red)

Severity score = 2 for *L. pneumophila* serogroups 2-14; severity score = 3 for serogroup 1 (SG1). Likelihood score is assigned directly from the presence-weight matrix (Table S2), where 0 = absent, 1 = occasional, 2 = regular, and 3 = frequent/usual. Color coding: green = low, yellow = moderate, red = high.

Table S4. Minimum task-based personal protective equipment (PPE) recommendations by worker group.

Worker Group / Task type	Gloves	Eye/Face Protection	Waterproof Clothing	Respiratory PPE (FFP2/FFP3)
Outlet flushing in contaminated rooms	✓	✓	✓	✓ (FFP2–FFP3)
Showerhead descaling	✓	✓	✓	✓ (FFP2–FFP3)
Opening mechanical rooms after stagnation	✓	✓	✓	✓ (FFP3)
Pool/spa filter backwashing	✓	✓	✓	✓ (FFP2–FFP3)
Ice machine or kitchen spray maintenance	✓	Optional	✓	Optional
Routine housekeeping	✓	–	–	–
Lifeguard/poolside work	–	–	–	–

Table S5. Availability of physicochemical measurements and proportion of deviations among recorded values for temperature (T_i), free residual chlorine (Cl_i), and pH (pH_i).

Criterion	Recorded (n)	Deviations (n)	Deviation % (of recorded)
Temperature deviation (T_i) (<55 °C)	552	426	77.17
Temperature deviation (T_i) (<50 °C)	552	344	62.32
Free chlorine deviation (Cl_i)	243	70	28.81
pH deviation (pH_i)	143	3	2.10

Table S6. Zone-level environmental hazard scores by functional area under the semi-quantitative environmental hazard model.

Functional area	Scored (n)	Mean $\bar{H}_z$	SD
Kitchens and Food & Beverage Areas	14	2.607	1.130
Machinery Rooms and Water Production/Storage Systems	90	2.022	1.503

Guest Rooms	405	1.874	1.404
Recreational Areas (Pools/Spa)	63	1.825	1.097
Gardens and Outdoor Plumbing/Irrigation	6	1.750	0.758

Table S7. Task-based personal protective equipment (PPE) recommendations for worker groups performing high-exposure or aerosol-generating activities in hotel water systems.

Worker Group	High-Risk Tasks	Minimum PPE Required
Maintenance technicians	Opening mechanical rooms after stagnation; flushing outlets; repairing hot-water circuits	Gloves, goggles, waterproof clothing, FFP2/FFP3 respirator
Plumbing/HVAC technicians	Pipework repairs; showerhead descaling; tank cleaning	Gloves, face shield, waterproof apron, FFP2/FFP3 respirator
Pool & spa technicians	Filter backwash, dosing adjustments, spa draining/refilling	Gloves, splash goggles, waterproof clothing, FFP2 respirator
Chemical dosing technicians	Handling chlorine/bromine dosing systems	Chemical-resistant gloves, goggles, waterproof apron, FFP2 respirator
Spa attendants	Operation of high-aerosol spa facilities	Gloves, eye protection
Lifeguards	Exposure near splash zones and pool showers	No routine PPE (unless assisting in maintenance)
Housekeeping staff	Cleaning showers; flushing outlets in contaminated rooms	Gloves, simple eye protection; FFP2 in confirmed contamination
Public area cleaners	Cleaning public showers and changing rooms	Gloves, eye protection
Kitchen staff	Dishwashing aerosol zones	Gloves; optional eye protection
Gardeners	Work near irrigation or misting systems	Gloves; eye protection if high-pressure systems operate
External plumbers/HVAC contractors	Same tasks as internal technical staff	Gloves, goggles, waterproof clothing, FFP2/FFP3 respirator
External pool/spa contractors	Spa and pool system interventions	Gloves, goggles, waterproof clothing, FFP2 respirator
Security/handyman	Minor repairs involving water outlets	Gloves
Technical safety specialists	Inspections in technical/high-risk areas	Gloves, basic eye protection
Public health inspectors	Sampling in contaminated/mechanical areas	Gloves, eye protection, FFP2 respirator
